# Longitudinal Effects of Parental Academic Support on Academic Achievement in Korea: Will You Be a Companion or a Manager in Your Children’s Academic Support?

**DOI:** 10.3390/ijerph182010823

**Published:** 2021-10-15

**Authors:** Yujin Jang, Youngmeen Suh

**Affiliations:** 1 Department of Early Childhood Education, Graduate School of Education, Catholic University of Korea, Bucheon-si 14662, Korea; yjjang@catholic.ac.kr; 2 Department of Early Childhood Education, College of Education, Mokwon University, Daejeon 35349, Korea

**Keywords:** parental academic support, self-regulation, amotivation, academic achievement

## Abstract

This study addresses the novel concept of two types of parental academic support (PAS), namely, as a companion and as a manager, and identifies the effect of children’s perceived PAS on their psychological attributes and academic achievements. The data include a nationally representative sample of 6836 students from the Korean Education Longitudinal Study 2013. A correlation analysis showed that the two types of PAS had a positive relation with adolescents’ development; however, a structural equation model showed a negative effect of PAS as a manager. Children’s perceived PAS as managers had no effect on their self-regulation or academic achievement after 3 years, surprisingly, was found to increase their amotivation. Children’s perceived PAS as companions had a long-term, positive effect on adolescents’ development and academic achievements. The results suggest that parents should recognize their children’s autonomy and provide academic support on an equal level. Further multidimensional PAS studies should be conducted with more detailed questions.

## 1. Introduction

Parental support for their children’s studies has been discussed regarding various concepts, such as academic support, emotional support, attention to achievement, parental involvement and school participation. Some studies show that parental support for children positively impacts their achievement [1,2]; however, other studies argue that excessive attention negatively impacts children [3,4]. According to a previous study, the emotional support of parents was positive for their children’s psychological health [5,6]. However, parents’ academic support and attention to achievement functions as a double-edged sword, as they affect positive motivation, predict negative motivation and test anxiety [7,8].

Parental academic support refers to the economic and environmental support provided by parents for their children’s successful academic achievement. Parental support, compared to teacher or peer support, predicts adolescents’ motivations, achievements and adjustment in various cultures [8,9]. Consistent results are readily available for parental emotional support; therefore, various studies on parental academic support (PAS) are needed. Song and colleagues’ study (2015) attempts to tease out the subtle differences between support and pressure as subjectively experienced by adolescent learners [8]. What causes conflicting results in PAS? Excessive PAS might be perceived as pressure. What is the difference between properly supporting and overly supporting a child? Most studies related to PAS did not clearly define excessive support.

In this study, we noted parents’ views of their children in considering PAS. Korea is a country with strong educational values and academic elitism, and Korean adolescents feel a strong sense of obligation to recompense their parents with high scholastic achievement for the support and the sacrifices that their parents have given them [8]. In the past, a child was considered a parent’s possession or an avatar, and the child had to obey the parent’s instructions [10]. Although many of these perceptions have recently disappeared in Korea, some parents do not think of their children as equal personalities because this patriarchal culture still exists. We considered that parents’ views of their children would also be shown in their academic support for them.

We examined parents’ roles in academic support regarding whether parents learn with the child as a companion or force the child to study as a manager. In addition, we discuss academic support and academic achievement in terms of process and motivation. Parenting affects not only children’s achievements [2,11] but also their motivation to achieve [7,12,13]. Parental time investment in children is important to their healthy development, both socially and psychologically [14,15]; that is, life patterns shaped in childhood carry strong effects on outcomes in adulthood [16,17].

The concept of “educational manager mothers” or “educational managers” is familiar in South Korea, and some studies have discussed this concept [18,19]. Managers are greatly involved in their children’s education with full control over their children’s daily routines and future plans for academic development [18], and they intensively devote time and energy to maximizing their children’s capacities to achieve future success. The “manager mother” is more like a mother who manages her child’s studies by efficiently organizing various information and knowledge, rather than a caring mother who unconditionally accommodates her child. Parents’ excessive management of their children’s studies is related to not recognizing a child as an independent being [10]. Therefore, as opposed to managers, we chose the word companion. PAS as a companion refers to the role of keeping an appropriate distance from children and supporting children without forcing them to bond with their parents in learning. Parents’ authority and control are challenged in their children’s late childhood and adolescence; therefore, parents must re-establish parent–child relationships [20]. In redefining the parent–child relationship, some people discuss the role of “parents as friends”. In particular, Korean children’s academic stress and the resulting suicide rate of adolescents are the highest among OECD countries. Given that children’s private education and academic support are mostly chosen by their parents [21], we believe that PAS as a companion has important meaning.

We surmise that parents who provide academic support as companions, that is, learning with and giving advice to their children, are likely to respect their children’s opinions and autonomy because they share the same position as their children. In contrast, parents who manage their children’s lives and academic grades are likely to force their will and thoughts on their children and to adopt a dominant position over their children rather than an equal level of control. They may attempt to control the psychology of their children by making them feel guilty or withdrawing parental affection when their children do not comply with their wishes. Parents’ autonomy support is related to children’s motivation and self-regulated academic efficacy among elementary school students [12,22,23], while parents’ psychological control negatively impacts children’s psychological health, including self-control, academic self-efficacy and helplessness [7,24].

These two types of PAS share similar aspects with parental autonomy support and psychological control. Parental autonomy support refers to parenting behaviour in which parents offer children the opportunity to select and set their own direction, which minimizes forceful evaluations, externally imposed objectives, and parental needs [10]. In contrast, psychological control refers to parental attempts of control that intrude on the children’s psychological and emotional development [25]. Psychological control seeks to influence children’s behaviour through covert strategies, such as guilt induction, withdrawal of love, invalidation of feelings, and creating an environment in which a parent’s acceptance of the child is contingent on the child’s behaviour [26,27].

These two approaches to PAS can be seen as similar to learning goals and performance goals, which affect subsequent human behaviour patterns according to motivational research. People with learning goals aim to learn new things and solve problems in learning situations. Therefore, students with learning goals tend to prefer difficult and challenging tasks and learn more effectively [28,29]. In contrast, people with performance goals aim to achieve positive results and avoid negative judgements about their abilities [30]. Therefore, it is easy for them to choose successful situations to prove their value, and children with performance goals are likely to be vulnerable to helplessness [31,32]. Parents who support their children as companion study together with their children and expect their children to acquire new information through a shared learning process. In contrast, parents who manage their children’s schedules and grades may be more interested in their children’s grades or achievements than in their learning processes.

According to the self-determination theory [33], one of the intrinsic motivation theories, people with a high self-determination and autonomy are willing to participate in challenges and autonomously perform difficult tasks according to their own interests and satisfaction. The theory suggests six types of human motivation depending on the degree of self-determination on a continuum from intrinsic motivation, which is the most autonomous and self-deterministic type of motivation, to amotivation, in which the person lacks motivation. There are four types of external motivation between intrinsic motivation and amotivation, which depend on the level of self-determination. Amotivation is one of the most negative types of motivation and adversely affects achievement and development. Amotivation is associated with school dropout intentions and negative academic achievements [34,35,36]. In some studies, amotivation is the only motivation type significantly associated with academic achievement [34,36]. Accordingly, amotivation is related to psychological well-being [37], and the degree of intrinsic motivation is related to the psychological health of students [38].

We expect that parenting, especially managerial academic support, will affect children’s amotivation. It is difficult to find a prior study that conceptualizes PAS in this way. However, as previously discussed, parents who support children’s studies as managers are likely to control their children’s autonomy, to control their children psychologically and to have performance goals. These characteristics were the variables that predicted children’s amotivation in prior studies. Schiffrin and Liss (2017) found that overinvolved parenting, such as helicopter parenting, was associated with extrinsic academic motivation, which may be adversely related to academic achievement [39]. Autonomous parenting types and intrinsic motivation were associated with positive academic results, better performance at school, and the most positive self-perceptions [40,41]. Similarly, amotivation and controlled forms of motivation were associated with negative consequences [34,36,42]. According to the self-determination theory [33], intrinsic and extrinsic motivation are not dichotomously distinguished, and different levels of extrinsic motivation occur depending on the level of self-determination. Therefore, it is necessary to understand the impact of the type of PAS and children’s amotivation towards academic achievement.

Self-regulation is an important variable that affects children’s academic achievement and development. Self-regulation, sometimes referred to as “self-monitoring or self-control”, is based on the principle of metacognition. Self-regulation is the ability to plan, check, evaluate, and control one’s own behaviour. It involves regulating the starting and stopping of actions and the intensity, frequency and persistence of activities in line with socially accepted behaviour in specific environments [43]. Accordingly, self-regulation is known to be related to the psychological and physical health of adults [44]. In an academic sense, self-regulation involves properly controlling one’s impulses and studying to achieve personal and academic goals. Children’s ability to regulate their behaviour in everyday life is a core skill for school life. Self-regulation skills directly impact a variety of positive academic outcomes, including improved homework completion and academic outcomes in the immediate future, and higher graduation rates and optimal college outcomes in the long run [45,46].

PAS is related to various aspects of children’s self-regulation and to academic self-regulation [47,48]. The support from parents, teachers, and peers directly or indirectly contributes to adolescent performance through adolescent motivation, especially parental support, which plays an important role in Korean adolescents’ motivation and grades [8]. Academic support in the school environment predicted students’ self-regulation, which predicted academic achievement, and self-regulation was mediated by adolescent motivation [49]. PAS is expected to affect children’s motivation and self-regulation, which ultimately affect their academic achievement.

## 2. Methods

The Korean Education Longitudinal Study (KELS) 2013 database included measures of parental support, self-regulation and achievement goals along with scores from the national standardized academic achievement test.

### 2.1. Participants

The data for this study are derived from the 2013 KELS conducted by the Korean Educational Development Institute (KEDI). The KELS began in 2013, and 8070 students from 242 schools were extracted from 524,117 fifth graders (born in 2002, 11 years old) who were attending 5509 elementary schools nationwide by using the stratified cluster random sampling method. Finally, 7324 students from 242 schools participated in the study, with a student sampling rate of 1.40% and a school sampling rate of 4.39%. Korea has a 6-3-3 education system, including 6 years of elementary school followed by 3 years each of middle and high school education. After promoting the survey to schools and parents, KEDI selected cooperative teachers and conducted a presentation about KELS. Of the 8070 students, 7324 who agreed to the study were used as final samples, and self-reporting surveys were conducted through structured questionnaires. The survey questions included a wide range of topics, such as school and home experiences and educational resources and support. The respondents will be resurveyed each year with different questionnaires until they reach the age of 28 in 2030. Surveys were administered separately to students and their families, teachers, and schools to collect a wide range of information.

This study used two out of four waves from the 2013 KELS, specifically, the first (5th grade) and fourth (middle school and 2nd grade) waves, which yielded a sample of 6836 cases (3369 boys, 49.28% and 3467 girls, 50.72%) that participated in all four waves and included responses arranged by students and their schools. Table 1 provides additional information on the parents’ ages and education levels.

### 2.2. Measures

#### 2.2.1. PAS

**Figure A1:**
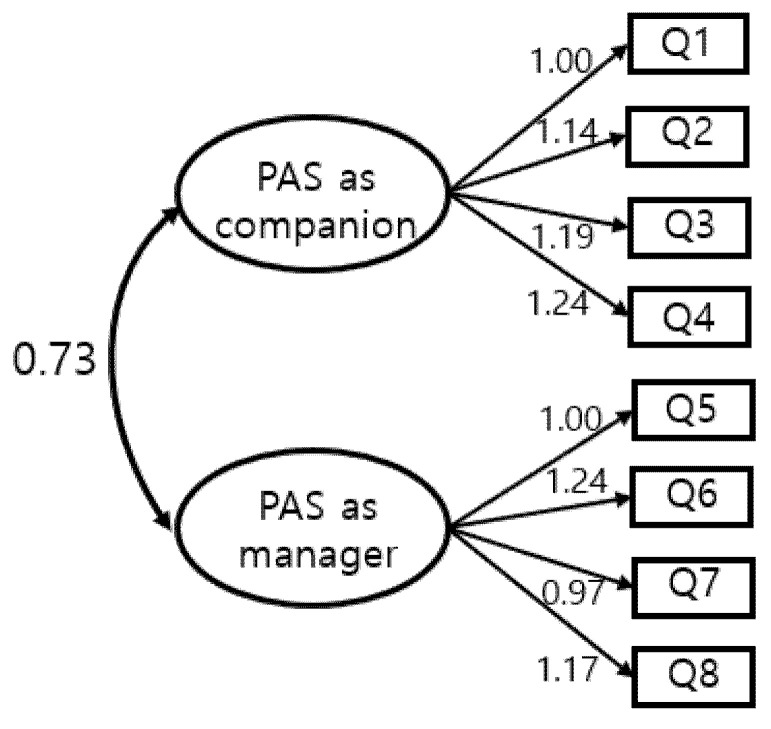
Confirmatory Factor Analysis of PAS. Notes. PAS, parental academic support; Q1–Q8, question1-question8.

PAS was measured with eight items that were classified by a factor analysis into four questions each (Table A1 and Figure A1). “PAS as companions” was used for the situation in which parents studied with children, taught children themselves, advised them how to study, and created a learning atmosphere at home. In contrast, the situation in which parents decided on children’s private tutoring or private educational institutes, spared no money for children’s studies, and managed children’s grades and schedules was named “PAS as managers”. The two types of PAS were coded on a 5-point (1–5) Likert scale, where each of the endpoints represented strongly disagree (1) and strongly agree (5). PAS as a companion was assessed by using four items, such as “My parents teach me how to study”, and PAS as a manager was assessed by using four items, such as “My parents collect information about private tutoring or private institutes for me”. The Cronbach’s α was 0.78 for PAS as a companion and 0.75 for PAS as a manager.

#### 2.2.2. Adolescents’ Psychological Attribute

In this study, the psychological attributes of children included self-regulation and amotivation. Psychological attributes were defined in various aspects, but this study aimed to discuss the psychological attributes related to academic achievement as a student. A mindset with a motivation for study, while properly regulating oneself for optimal academic achievement, was defined as psychologically healthy. Self-regulation was measured with five items and referred to academic self-regulation, such as “I do not put off what I have to do today”. Amotivation also consisted of four items that measured amotivation towards school or studies, such as “I do not know what I’m doing at school” (Appendix A). Psychological attribute was coded on a 5-point (1–5) Likert scale from strongly disagree (1) to strongly agree (5). The Cronbach’s α was 0.73 for self-regulation and 0.93 for amotivation.

#### 2.2.3. School Achievement

School achievement consisted of Korean, mathematics, and English scores and was measured by tests. This measure was developed based on item response theory (IRT) on a common scale (vertical scale) to compare the scores by grade in an attempt to interpret the changes in test scores for each grade as ability levels changed. The vertical scale was developed by applying a multigroup concurrent parameter estimation method. The theoretical achievement parameter was then linearly converted to an average of 200 points and a standard deviation of 40 points based on the fifth grade of elementary school.

### 2.3. Statistical Analyses

The descriptive statistics of the major variables, such as the mean, standard deviation, skewness, and kurtosis, were confirmed. The Cronbach’s α was presented to confirm the internal consistency of the scales. The descriptive statistics, Cronbach’s α coefficients, Pearson correlation coefficients and factor analyses were conducted with the SPSS 26 program. A factor analysis (principal components analysis) with a Varimax rotation was used to classify PAS (Appendix A and Appendix B). We assumed that PAS preceded adolescents’ psychological attributes, including self-regulation and amotivation, related to their academic achievement. We expected that the two types of PAS perceived in childhood, namely, PAS as a companion and PAS as a manager, directly affected the academic achievements of adolescents with a mediating influence of self-regulation and amotivation. To this end, a structural equation analysis was conducted with AMOS 21, and a maximum likelihood estimation (MLE) was used to estimate the parameters.

## 3. Results

### 3.1. Descriptive Statistics for the Main Variables

The means and standard deviations of the main variables are presented in Table 2. The mean score for PAS as a companion was 3.26 (SD = 0.74) and for PAS as a manager was 3.47 (SD = 0.90). First-year children’s perceived PAS as a manager score was somewhat higher than their PAS as a companion score. Adolescents’ self-regulation (M = 3.36, SD = 0.61) and amotivation (M = 2.10, SD = 0.59) were slightly positive. For academic achievement (standard score; M = 200, SD = 40), the Korean score was the lowest, and the English score was the highest. The distribution percentiles of each level of Korean were deficient at 6.5%, basic at 37.3%, normal at 42.3%, and proficient at 13.9%. The distribution percentiles for Math were deficient at 13.9%, basic at 33.2%, normal at 32.4%, and proficient at 20.4%. Finally, the distribution percentiles for English were deficient at 11.6%, basic at 15.6%, normal at 32.0%, and proficient at 40.8%.

### 3.2. Correlations between PAS and Adolescents’ Psychological Attribute and Academic Achievement

The results of the correlations between PAS and adolescents’ psychological attributes and academic achievements can be found in Table 3. Both PAS as a companion and PAS as a manager were correlated with adolescents’ psychological attributes and academic achievement. Furthermore, the subfactors of PAS were highly related to one another (r = 0.55, *p* < 0.000). PAS was positively related to adolescents’ self-regulation and academic achievement. However, PAS was negatively related to adolescent amotivation. The findings suggest a high probability that PAS can positively affect adolescents’ development.

The subfactors of PAS showed the same aspects of adolescents’ psychological attribute and academic achievement. Both PAS as a companion and PAS as a manager showed correlations with adolescents’ self-regulation and Korean, Math, and English grades; however, both types of PAS showed a negative correlation with amotivation. The correlation coefficients differed, and the coefficients were generally higher for PAS as a companion than for PAS as a manager, except for Math (PAS as a companion: r = 0.14, PAS as a manager: r = 0.15). It is most likely possible that both PAS as a companion and PAS as a manager might have a positive effect on children’s development or academic achievement.

### 3.3. Effect of PAS and Adolescents’ Psychological Attribute on Academic Achievement

**Figure 1:**
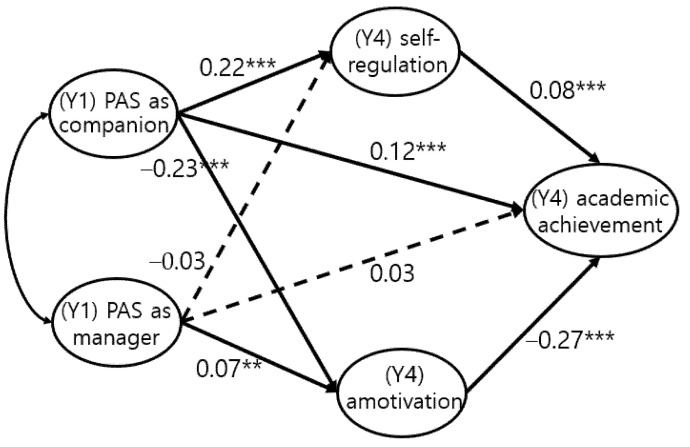
Final Model: The effect of PAS on academic achievement mediated by self-regulation and amotivation. ** *p* < 0.01, *** *p* < 0.001.

A structural equation analysis was conducted on the assumption that PAS would affect adolescents’ psychological attributes and academic achievements, and that adolescents’ psychological attributes would affect their academic achievements. The model was a good fit for the data. The coefficients are presented in Figure 1 and Table 4.

However, the structural equation model showed quite different results. PAS as a companion had a positive effect on adolescents’ self-regulation and academic achievements and a negative effect on their amotivation. PAS as a manager, however, did not affect adolescents’ self-regulation and academic achievements and had a positive effect on amotivation. Self-regulation had a positive effect on academic achievement, while amotivation had a negative effect. PAS as a manager, which strives for young children’s academic achievements, did not affect children’s self-regulation or academic achievement three years later but instead increased motivation. In contrast, children who perceived PAS as a companion in the first year attained a high academic achievement by having high levels of self-regulation and low levels of amotivation.

## 4. Discussion

This study was conducted to identify the effects of children’s perceived PAS on the psychological attributes of adolescents and their academic achievements. Unlike previous studies, this study classified PAS into two types through a factor analysis and identified the different effects of each type of PAS on adolescents. PAS was divided into PAS as a companion and PAS as a manager. PAS as a companion is parental support such as studying with children, teaching children personally, advising them how to study, and creating a study-friendly environment at home. PAS as a manager is parental support such as deciding on children’s private tutoring or private educational institutes, sparing no money for children’s studies, and managing children’s grades and schedules. In this study, we discussed more desirable parental roles for children’s academic support.

Children’s perceptions of PAS as both companions and managers were correlated with adolescents’ psychological attributes and academic achievements. In this study, PAS had a positive relation with adolescents’ development and academic achievements. However, the two types of PAS had different correlation coefficients, although the directions of the correlation were the same. This occurred because the two types of PAS were originally one concept. That is, the concepts of PAS as a companion and PAS as a manager were not contradictory; although they had different characteristics, it was reasonable to assume that they shared certain aspects. According to prior research [50,51], external motivation due to parental pressure could also be positive, especially for Asian students, which was the context for this study’s findings.

The structural equation model analysis results were striking. The correlation analysis showed that the two types of PAS had a positive relationship with adolescents’ development; however, the structural equation model showed a negative effect of PAS as a manager. Children’s perceived PAS as a companion positively affected adolescent self-regulation and academic achievement and reduced amotivation. However, children’s perceived PAS as a manager had no effect on adolescent self-regulation and academic achievement and surprisingly, was shown to increase amotivation. Amotivation had a significant negative impact on academic achievement. In the same context, parents’ attention to achievement increased children’s academic self-efficacy; however, parents’ attention to achievement did not affect children’s motivation [7], which was predicted to originate from parents’ attention to achievement being associated with their psychological control. We conclude that coercive parenting that violates children’s autonomy and motivation negatively affects children’s motivation and ultimately adversely affects children’s academic achievements. If children feel that their parents manage them but do not accompany them at 8 years old, then they are likely to have amotivation after three years, and amotivation negatively affects adolescents’ academic achievement. Therefore, parents may reduce children’s helplessness response by improving academic self-efficacy through autonomy-supportive parenting rather than by increasing their focus on children’s academic achievements.

Through this study, we confirmed that PAS as a companion has a long-term and positive impact on adolescents’ development and academic achievements. Parents should not support their children’s studies by acting as a manager of their children’s time and grades but by learning with and accompanying their children. Parents who study with their children and support their studies as companions understand and support their children’s interests, preferences, and perspectives more than parents who support their children as managers. PAS as companion parenting may increase children’s autonomy. This result is explained by considering that parental social support affects children’s motivations and achievements [8]. In addition, parents who support their children provide consistent, reasonable expectations and structures that consider their children’s situation, and their children have the opportunity to judge for themselves what they can and cannot do. The accumulation of these experiences positively impacts academic achievement by encouraging children to develop the ability to regulate themselves and their intrinsic motivation. This type of parenting is similar to the use of learning goals, which value the process over the outcome of learning. In addition, PAS as a companion parenting is similar to “autonomy-supportive parenting”, which refers to parenting behaviour in which parents not only offer children the opportunity to select and set their own direction but also minimize forceful evaluations, externally imposed objectives, and parental needs [24]. Autonomy-supportive parenting accommodates children’s perspectives and helps them to develop their own interests and personal values [52].

When children perceived that their parents managed their grades, schedules and private education, their level of adolescent amotivation was higher. Predictably, this perception increased the likelihood that children would expect their parents to manage their studies even if they did not manage them themselves. Furthermore, these adolescents may have a reduced motivation and willingness to learn and control themselves. Children who experience negative reactions to their independent and autonomous endeavours rely on external approval, such as parental approval or management, and tend to follow externally set goals, thereby reducing their motivation to act autonomously. Parental support as a manager does not directly affect adolescents’ self-regulation; however, it does affect adolescents’ amotivation. For adolescents, amotivation negatively affects their academic achievement, which aligns with the findings that autonomy control and intrusive parenting negatively impact children’s development and academic achievements [7,53,54].

Humans are autonomous beings, and autonomy is very important in academic aspects. It is desirable for parents to stand by their children, create a learning environment and be an advisor to help their children when they are in trouble, rather than managing children’s schedules and private education. It is important when supporting children’s studies to respect their autonomy, and parents and children should be on an equal footing. Even if the child is young, the role of a parent as a companion is important, and has a longitudinal effect. This study discusses the importance of the parental role as a companion in supporting and strengthening children’s motivation and self-regulation. When parents learn and grow together while respecting their children’s will and coordinating their opinions, children will be able to achieve their optimal development. Parents are the strongest source of perceived support for adolescents [8]. Children can have optimal self-regulation, motivation, and academic achievement in the psychologically autonomous and safe environments that their parents provide.

Although this study has the advantage of having a large number of subjects due to the use of panel data, there was a limit to the number and content of the variable items. Specifically, more items are needed to discern the PAS type. In further studies, the two types of PAS (PAS as a companion and PAS as a manager) must be more clearly discussed and defined by a factor analysis with more questions. Due to the large number of cases, some variables were statistically significant, despite the low correlation coefficient. In the future, various studies and discussions that use these variables are needed. Moreover, some of the relationships associated with parents might be stronger in Korean children than they would be in Western samples because of sociocultural factors specific to the Korean context. Future studies may require the use of samples from various cultures.

## Figures and Tables

**Table 1:** Parents’ Demographics: Frequency and Percentage of Age and Education Level.

Variables		Father *n* (%)	Mother *n* (%)
Age	24–39 years	882 (13.34)	2589 (37.87)
	40–49 years	4566 (69.10)	3946 (57.72)
	50–59 years	444 (6.72)	151 (2.21)
	No answer	716 (10.84)	150 (2.19)
Education level	High school and below	2402 (36.35)	3094 (45.26)
	Community college	1142 (17.28)	1456 (21.30)
	Four-year university	2114 (31.99)	1893 (27.69)
	Graduate school and above	651 (9.85)	290 (4.24)
	No answer	299 (4.52)	103 (1.51)
Total		6608 (100.0%)	6836 (100.0)

**Table 2:** Descriptive Statistics for the PAS, Adolescent Psychological Attribute and School Achievement.

	Variables		M (SD)	Range	Skewness	Kurtosis
Year 1	PAS	as companion	3.26 (0.74)	1–5	−0.12	−0.27
		as manager	3.47 (0.90)	1–5	−0.21	0.08
Year 4	Adolescent psychological attribute	self-regulation	3.36 (0.61)	1–5	−0.04	0.16
		amotivation	2.10 (0.59)	1–5	0.84	0.39
	School achievement (test scores)	Korean	230.09 (46.34)	140–329	−0.20	−0.82
		Math	231.67 (50.96)	147–324	0.08	−1.06
		English	250.16 (51.51)	150–331	−0.14	−1.00

Notes. M, mean; SD, standard deviation; PAS, parental academic support.

**Table 3:** Correlations of PAS with Adolescents’ Psychological Attribute and School Achievement.

	PAS (Y1)		Psychological Attribute (Y4)		Academic Achievement (Y4)		
	1. As Companion	2. As Manager	3. Self-Regulation	4. Amotivation	5. Korean	6. Math	7. English
1	1						
2	0.55 ***	1					
3	0.16 ***	0.12 ***	1				
4	−0.14 ***	−0.09 ***	−34 ***	1			
5	0.10 ***	0.04 **	0.15 ***	−0.24 ***	1		
6	0.14 ***	0.15 ***	0.16 ***	−0.25 ***	0.63 ***	1	
7	0.17 ***	0.14 ***	0.19 ***	−0.26 ***	0.69 ***	0.70 ***	1

** *p* < 0.01, *** *p* < 0.001 Correlation matrix table including the Pearson correlation coefficient (R). PAS, parental academic support; Y1, year 1; Y4, year 4.

**Table 4:** Path Coefficients of PAS and Adolescent Psychological Attribute.

Path	Estimate (S.E.)	Standardized Estimate
PAS as companion ⇒ self-regulation	0.19 (0.02) ***	0.22
PAS as companion ⇒ amotivation	−0.25 (0.03) ***	−0.23
PAS as manager ⇒ self-regulation	−0.02 (0.03)	−0.03
PAS as manager ⇒ amotivation	0.08 (0.03) **	0.07
self-regulation ⇒ academic achievement	3.71 (0.75) ***	0.08
amotivation ⇒ academic achievement	−10.14 (0.52) ***	−0.27
PAS as companion ⇒ academic achievement	4.73 (1.09) ***	0.12
PAS as manager ⇒ academic achievement	1.54 (1.23)	0.03

Model Fit; χ^2^ = 4467.06, df = 161, χ^2^/df = 27.75, *p* = 0.000, NFI = 0.93, IFI = 0.93, CFI = 0.93, RMSEA = 0.06. Notes. S.E, standard error; df, degree of freedom; NFI, normed fit index; IFI, incremental fit index; CFI, comparative fit index; RMSEA, root mean square error of approximation; PAS, parental academic support. ** *p* < 0.01, *** *p* < 0.001.

## Data Availability

Data from the Korean Education Longitudinal Study of 2013 are not publicly available. If you require the data, then complete and submit the data application and the data will be provided through KEDI’s review. See https://www.kedi.re.kr/khome/main/research/requestResearchData.do (accessed on 1 March 2021) for obtaining access to the data.

## Appendix A

**Table A1:** Factor Loading of Items.

Variables and Items	*M (SD)*	Factor Loadings
Parental academic support (PAS) as companion		
My parents teach me how to study.	2.83 (1.22)	0.83
My parents give me advice on how to study.	3.40 (1.17)	0.80
My parents provide me with a study-friendly environment.	3.51 (1.06)	0.72
My parents check my schoolwork and homework.	3.32 (1.15)	0.66
Parental academic support (PAS) as manager		
My parents collect information about private tutoring or private institutes for me.	3.21 (1.22)	0.82
My parents spare no money for my studies.	3.59 (1.09)	0.75
My parents care about my grades.	3.55 (1.10)	0.65
My parents check in about my daily life and manage my schedule.	3.53 (1.07)	0.62
Self-regulation		
I do not put off what I have to do today.	3.09 (0.96)	0.78
I organize and clean my desk by myself.	3.71 (0.99)	0.76
If I have a lot of work to do, I make a plan and complete tasks one-by-one.	3.37 (1.05)	0.75
I pack the supplies I need to bring to school the next day by myself.	4.01 (0.85)	0.70
I often watch TV or play before doing my homework.	2.61 (1.05)	0.47
Amotivation		
I do not know what I’m doing at school.	1.99 (1.04)	0.93
I do not know why I go to school.	2.03 (1.03)	0.92
I feel like I’m wasting my time at school.	2.20 (1.08)	0.90
I do not know why I have to study.	2.20 (1.03)	0.90

Notes. Factor analysis of measures, KMO = 0.863, Bartlett test, χ^2^ = 38,987.07, df = 136, *p* = 0.000. M, mean; SD, standard deviation; KMO, Kaiser–Meyer–Olkin.
